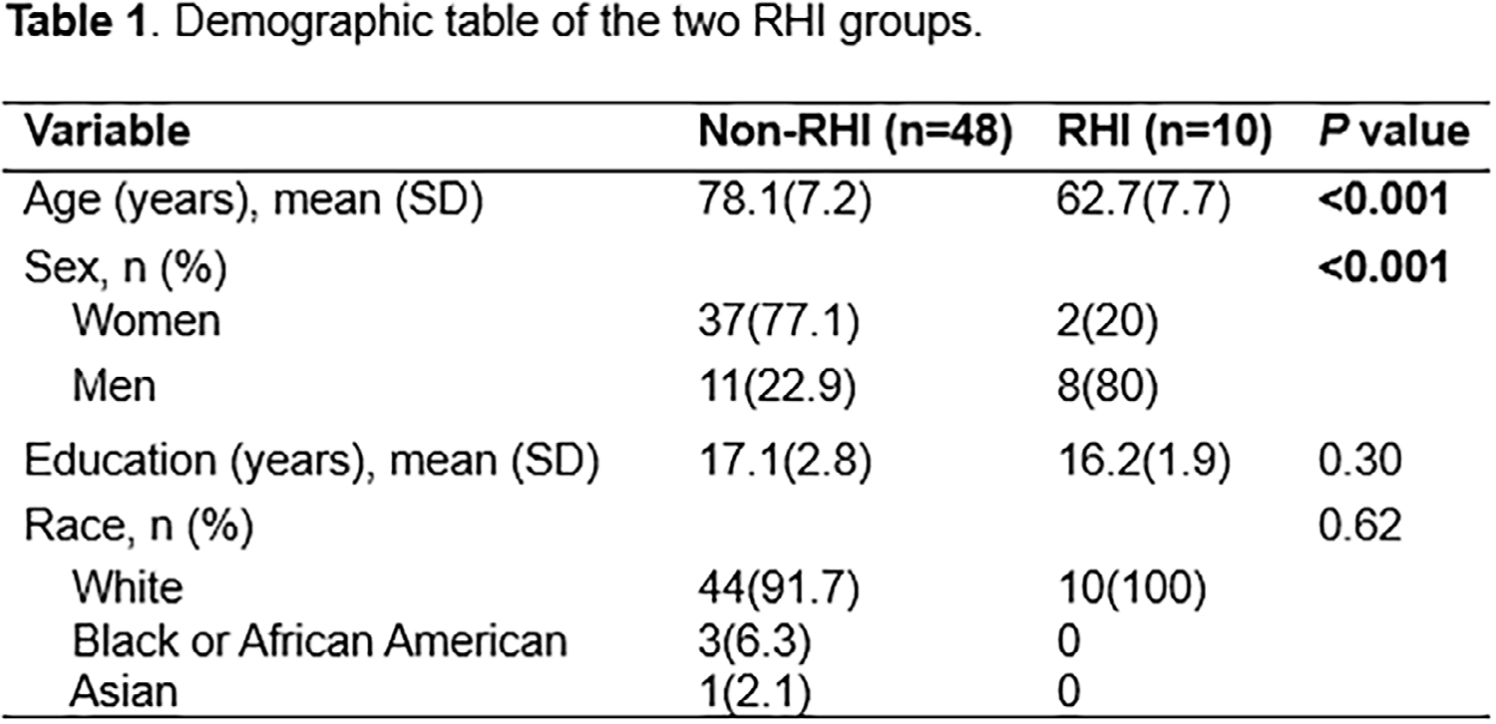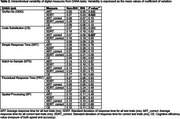# Exploring the Effect of Repetitive Head Impacts on the Stability of Digital Cognitive Measures in Cognitively Intact Older Adults: A Pilot Study

**DOI:** 10.1002/alz70856_103876

**Published:** 2025-12-26

**Authors:** Huitong Ding, Xavier Serrano, Edward Searls, Kristi Ho, Zexu Li, Alexa Burk, Margaret Low, Chenglin Lyu, Katherine A. Gifford, Vijaya B. Kolachalama, Honghuang Lin, Christopher J. Nowinski, Joseph N. Palmisano, Yorghos Tripodis, Katherine W. Turk, Andrew E. Budson, Ann C. McKee, Jesse Mez, Rhoda Au, Michael L. Alosco

**Affiliations:** ^1^ Boston University Chobanian & Avedisian School of Medicine, Boston, MA, USA; ^2^ Dept of Anatomy & Neurobiology, Boston University Chobanian & Avedisian School of Medicine, Boston, MA, USA; ^3^ University of Massachusetts Medical School, Worcester, MA, USA

## Abstract

**Background:**

Exposure to repetitive head impacts (RHI) from contact and collision sports, military service, and other sources have been linked to cognitive decline and neurodegenerative disorders. Traditional neuropsychological measures often miss the subtle cognitive deficits that can occur in individuals exposed to RHI. Digital measures might be more sensitive alternatives. However, little is known about how RHI influences the stability of digital neuropsychological measures in cognitively intact older adults.

**Method:**

This pilot study analyzed data from participants enrolled in the Boston University Alzheimer's Disease Research Center. RHI status is based on the 2021 NINDS TES Research Diagnostic Criteria. Participants complete annual study visits that includes Uniform Data Set. In 2021, Defense Automated Neurobehavioral Assessment (DANA) was introduced. The sample included cognitively intact older adults with at least three days of engagement with DANA. Intraindividual variability was quantified using the coefficient of variation (CV), and Mann‐Whitney U tests compared CVs between RHI and non‐RHI groups. Day‐to‐day variations and group differences were further examined with a linear mixed‐effects model, adjusting for age, sex, the number of usage days and RHI group, plus their interaction term.

**Result:**

This study included 58 participants (mean age: 75.4±9.3 years; 67.2% women; mean education: 17±2 years; 93.1% White) (Table 1). Of these, 10 were RHI, with soccer as the primary sport for 4, football for 2, rugby for 1, and other sports for 3. Overall, day‐to‐day fluctuations in DANA performance were small, yet individuals with RHI showed significantly greater variability across some tasks (e.g., Go/No‐Go, Code Substitution, Simple Response Time) than those without RHI (Table 2). Notably, in the Procedural Response Time task, each additional day of DANA usage was significantly associated with a 21.6 ms faster average response time for all test trials (*P* = 0.007). However, a significant interaction effect (beta = 38, *P* = 0.012) was observed for the RHI group.

**Conclusion:**

Although digital measures were generally stable across days, older adults with RHI exhibited more pronounced variability and appeared to benefit less from repeated practice. These findings underscore the need to consider RHI history when interpreting longitudinal cognitive assessments in aging populations.